# Increased risk of leukaemia in children with Down syndrome: a somatic evolutionary view

**DOI:** 10.1017/erm.2021.6

**Published:** 2021-04-27

**Authors:** K. A. L. Hasaart, E. J. M. Bertrums, F. Manders, B. F. Goemans, R. van Boxtel

**Affiliations:** 1 Princess Máxima Center for Pediatric Oncology, Heidelberglaan 25, 3584 CS Utrecht, the Netherlands; 2 Oncode, Utrecht, the Netherlands; 3 Department of Pediatric Oncology/Hematology, Erasmus Medical Center, Doctor Molewaterplein 40, 3015 GD, Rotterdam, the Netherlands

**Keywords:** Down syndrome, DS-ALL, ML-DS, paediatric leukaemia, somatic evolutionary model, TAM

## Abstract

Children show a higher incidence of leukaemia compared with young adolescents, yet their cells are less damaged because of their young age. Children with Down syndrome (DS) have an even higher risk of developing leukaemia during the first years of life. The presence of a constitutive trisomy of chromosome 21 (T21) in DS acts as a genetic driver for leukaemia development, however, additional oncogenic mutations are required. Therefore, T21 provides the opportunity to better understand leukaemogenesis in children. Here, we describe the increased risk of leukaemia in DS during childhood from a somatic evolutionary view. According to this idea, cancer is caused by a variation in inheritable phenotypes within cell populations that are subjected to selective forces within the tissue context. We propose a model in which the increased risk of leukaemia in DS children derives from higher rates of mutation accumulation, already present during fetal development, which is further enhanced by changes in selection dynamics within the fetal liver niche. This model could possibly be used to understand the rate-limiting steps of leukaemogenesis early in life.

## Introduction

Down syndrome (DS) is the most common chromosomal disorder and has a prevalence of approximately one in every 700 live births worldwide (Refs 1, 2). DS is caused by a full trisomy of chromosome 21 (T21) in 90% of the cases (Ref. 3). Nevertheless, other chromosome 21 abnormalities also cause DS, such as partial T21, translocations involving chromosome 21 and mosaic T21 (Refs 3, 4). The DS-related clinical phenotypes involve multiple body systems, in particular the neurological, cardiovascular and musculoskeletal systems (Ref. 5). These typical DS-phenotypes are believed to result directly from the extra copy of human chromosome 21 (Ref. 6). Studies performed in DS individuals with partial T21 suggest that a critical region of this chromosome (DS critical region; DSCR) is responsible for these phenotypes (Ref. 6).

DS individuals show a unique cancer distribution pattern during life (Refs 3, 7, 8). They have an increased risk of developing leukaemia during the first years of life and a marginal increased risk of developing germ-cell tumours, but show a decreased risk of solid tumours throughout life (Refs 8–10). DS children have a 500-fold increased risk of developing myeloid leukaemia of DS (ML-DS), a subtype of acute megakaryoblastic leukaemia (AMKL) (Ref. 9). ML-DS is often preceded by transient abnormal myelopoiesis (TAM) (Ref. 11), which occurs in 5–30% of all neonates with DS, depending on the diagnostic criteria that are used (Refs 12, 13). TAM is characterised by circulating myeloid leukaemic blasts in peripheral blood, harbouring mutations in *GATA1* (Refs 12, 13). TAM spontaneously disappears within the first 3 months after birth; however, 20% of these patients subsequently develop ML-DS before the age of five years (Refs 13, 14). ML-DS in these children is characterised by the same unique *GATA1* mutation that occurred in the (pre)leukaemic TAM blasts as well as additional oncogenic mutations (Ref. 15). In addition, the risk of acute lymphoblastic leukaemia (ALL) during the first years of life is increased 7–20 times compared with non-DS children (Refs 3, 9, 12). These leukaemias consist almost exclusively of B-cell precursor ALL (BCP-ALL) (Refs 3, 9, 12). Both DS acute lymphoblastic leukemia (DS-ALL) and ML-DS present with different genetic aberrations compared with their non-DS counterpart (Refs 16–18). The genetically and clinically distinct characteristics of TAM, ML-DS and DS-ALL compared with other paediatric leukaemias raise the question of why children with DS have an increased risk of developing leukaemia during their first years of life.

In this review, we provide a comprehensive overview of available literature and describe the increased risk of leukaemia in DS as a process of Darwinian evolution occurring among cell populations (Ref. 19). In this model, inheritable phenotypic diversity between cells is the substrate of selective forces driven by specific ecological features present within tissues (Ref. 19). Somatic mutations that accumulate throughout life as well as epigenetic changes can drive this phenotypic diversity. Ultimately, oncogenic mutations can provide a cell with a growth advantage, which in the correct tissue context results in clonal expansion and eventually cancer (Ref. 19). We hypothesise that the phenotypic diversity of cells, which is already present during fetal development (Refs 20–22), initiates leukaemic development in DS. Subsequent progression towards full-blown leukaemia is then fuelled by changes in selection dynamics of the niche as the site of haematopoiesis migrates from the liver to the bone marrow. Studies have shown that non-DS paediatric leukaemia often has an *in utero* origin as well, since mutations driving paediatric leukaemia have been detected in neonatal bloodspots (Ref. 23). If this model can indeed be used to explain the high risk of leukaemia in DS, then this could potentially be extended to understand the rate-limiting steps of leukaemogenesis in general early in life.

## Heritable phenotypic diversity in haematopoietic stem cell pools

Cell-intrinsic mutational processes and exogenous mutagenic exposures cause DNA damage, which will result in somatic mutations if the damage is not efficiently or incorrectly repaired (Ref. 24). Although DNA repair pathways are highly efficient, a part of the damage escapes these mechanisms, resulting in a gradual accumulation of somatic mutations throughout life (Refs 22, 25). This accumulation of mutations with age may explain why ageing is the biggest risk factor for developing cancer (Ref. 26). Indeed, the higher the number of somatic mutations in the genome of a cell, the higher the chance that one of these genetic alterations may serve as an oncogenic event. In DS children, T21 serves as the first genetic driver for leukaemic development (Refs 12, 27). Importantly, T21, either complete or partially, is one of the most frequent chromosomal alterations found in paediatric B-ALL and is often seen in non-DS AMKL (Refs 27, 28). Moreover, two percent of all paediatric B-ALLs show an intra-chromosomal amplification of chromosome 21 (iAMP21), which overlaps largely with the DSCR. This overlap stresses the importance of T21 and the DSCR as an initiation event in leukaemogenesis (Ref. 27). However, additional drivers are required for the initiation of leukaemogenesis (Ref. 12). Besides oncogenic mutations, also epigenetic changes, such as alterations in DNA methylation and chromatin modifications, are known to be associated with DS-associated leukemogenesis (Ref. 29).

### Genetic drivers of progression towards TAM and ML-DS

T21 in combination with a somatic truncating mutation in *GATA1*, acquired prenatally, is required and sufficient for the development of TAM (Refs 30–32). *GATA1* is located on the X-chromosome and encodes a haematopoietic transcription factor with essential functions for the differentiation of haematopoietic stem cells (HSCs) towards the erythroid and megakaryocytic lineages (Ref. 33). Most mutations are found in exon 2 of *GATA1* and result in an N-terminally truncated protein isoform GATA1s because of the utilisation of an alternative start codon located in exon 3 (Refs 2, 15, 32, 34). GATA1s lacks the amino-terminal activation domain and has a reduced transactivation potential, which causes decreased expression of *GATA1* target genes (Ref. 14). Although the DNA binding domain of GATA1s is intact, GATA1s binding is impaired at specific erythroid regulatory regions and the *MYC* promoter, whereas binding to megakaryocytic and myeloid target genes is normal (Refs 35–38). Also, studies have shown that GATA1s is not able to bind to RUNX1, an important protein for megakaryocyte differentiation; however, evidence is inconsistent (Ref. 39). As a result, GATA1s in T21 causes aberrant differentiation and proliferation of megakaryocytes, which results in the production of megakaryoblasts (Refs 6, 40). In addition, the expression of GATA1s is increased by the extra copy of the 4 megabase DSCR containing *RUNX1, ETS2* and *ERG*, which further promotes the proliferation of megakaryoblasts (Refs 6, 40).

Upon progression to ML-DS later in life, the same *GATA1* mutations are present, originating from a dominant or minor TAM clone, which indicates that ML-DS evolves from a persistent TAM clone during clinical remission (Refs 15, 33, 41). Nonetheless, additional driver mutations are required for progression towards ML-DS (Refs 15, 30). Thus far identified additional drivers include mutations in cohesins (53%) or *CTCF* (*20%*), *EZH2, KANSL1* and other epigenetic regulators (45%) and common signalling pathways including *JAK* family kinases, *MPL*, *SH2B3*, *CSFR2B* and multiple *RAS* pathway genes (47%), which all provide the cell with a growth advantage (Refs 30, 31, 41). Cohesins are essential for DNA replication and repair and are thought to have a tumour-suppressor function in cancer (Refs 42, 43). Models of dose-specific cohesin loss have shown HSC expansion and impairments in differentiation as a result of an open-chromatin state, which causes increased transcription of genes involved in self-renewal (Refs 42, 43). Mutations in epigenetic regulators and *CTCF*, which directly interacts with cohesins, are known to disrupt the expression of genes involved in HSC renewal and differentiation by chromatin modifications (Refs 44, 45). Both the JAK-STAT pathway and RAS signalling pathway are involved in the regulation of cell proliferation, survival and differentiation (Refs 46, 47). Mutations in these genes have been identified in different haematological malignancies and have been shown to contribute to the proliferation of leukaemic blasts (Refs 46, 47). In conclusion, mutations in these genes could all result in increased cell survival, cell proliferation and impairments in the differentiation of the oncogenic clone (Refs 42, 46, 47).

Mutations in cohesins, *CTCF* and *EZH2* showed a similar variant allele frequency (VAF) to *GATA1* mutations, which indicates that they are the oncogenic mutations driving the progression towards ML-DS (Ref. 30). On the other hand, the RAS pathway, other tyrosine kinases and cytokine receptor mutations had a lower VAF compared with *GATA1* mutations, indicating they originate from a later timepoint (Ref. 30). These varying VAFs point towards the subclonal development of ML-DS (Ref. 30). In rare cases, some of the driver mutations identified in ML-DS were present at a low VAF in the (pre)leukaemic TAM blasts (Ref. 41). This observation supports the hypothesis that in some cases the ML-DS clone has been already present early in life, possibly prenatal. This hypothesis is further supported by a study of monozygotic twins who share the same *GATA1* mutation and somatic translocation involving *CUX1*, identified at the time of simultaneous ML-DS development (Ref. 48).

The mutations driving ML-DS have, at lower frequencies, also been identified in non-DS AMKL (Refs 30, 49), indicating that ML-DS has a different mutational landscape compared with paediatric non-DS AMKL. Non-DS AMKL frequently presents with specific fusion genes as driving aberrations, such as *CBFA2T3*-*GLIS2* and *RBM15*-*MKL1*, which both have not been detected in TAM and ML-DS (Refs 30, 49). On the other hand, somatic T21 is frequently seen in children with non-DS AMKL, underlining the role of an extra chromosome 21 in leukaemic development (Ref. 28). Interestingly, these AMKL blasts frequently carry a somatic *GATA1* mutation and the same spectrum of additional aberrations as seen in ML-DS (Ref. 50).

### Genetic drivers of progression towards ALL

DS-ALL patients show a different spectrum of cancer driver gene mutations compared with non-DS ALL patients (Refs 16–18, 51). High hyperdiploidy, *ETV6-RUNX1* and *BCR-ABL1* are less common in DS-ALL compared with non-DS ALL (Ref. 18). Up to 62% of all DS-ALL cases show upregulation of *CRLF2*, caused by rearrangements (i.e. *IGH-CRLF2, P2RY8-CRLF2*) or mutations in *CRLF2,* compared with 5–12% of non-DS ALL cases (Refs 16, 17, 51, 52). Other cancer initiation events in DS-ALL are mutations in chromatin remodelers, classic tumour suppressors and B-lymphocyte differentiation factors (Ref. 17). Moreover, DS-ALL displays an increased incidence of activating *JAK2* mutations, which are only found in *CRLF2* overexpressing cases (Refs 16, 17, 51, 53). This connection suggests a cooperating effect of these genes in leukaemogenesis and *CRLF2* upregulation as a potential first event, before additional genetic aberrations in *JAK2* (Ref. 51). CRLF2 is an atypical type I cytokine receptor and a weak activator of *JAK2*, and thus the JAK-STAT pathway (Ref. 51). Hence positive regulation of *CRLF2* by *JAK2* may partially explain the identified relation (Ref. 51). CRLF2 binds the ligand TSLPR, which promotes early B-cell development (Refs 54, 55). Upregulation of *CRLF2* likely causes increased proliferation of early B-cells. Gain-of-function mutations have also been identified in *IL7R*, which forms a heterodimeric complex with CRLF2 (Refs 55, 56). *IL7R* is required for normal lymphoid development and part of the JAK-STAT signalling pathway, which further stresses the importance of the JAK-STAT pathway in DS-ALL (Refs 17, 55, 56). DS-ALL patients more often harbour *KRAS* and *NRAS* oncogenic mutations compared with non-DS ALL patients, which are mutually exclusive with *JAK2* mutations (Ref. 17). Interestingly, a complete or partial gain of chromosome 21 is often seen in non-DS paediatric B-ALL cases, predominantly in hyperdiploid ALL and iAMP21 ALL (Refs 27, 57). Similar to in DS-ALL, *NRAS* and *KRAS* mutations are often found in these patients, suggesting that T21 selects for these specific mutations (Refs 58, 59). However, the mechanism behind this cooperation is yet unknown.

### Somatic mutation accumulation during fetal development

As explained before, besides T21, additional oncogenic mutations are needed for leukaemogenesis in DS (Ref. 31). Since TAM is already present in DS newborns, mutation accumulation has to occur before birth (Ref. 60). In some TAM cases, multiple *GATA1* mutated clones have been detected, characterised by distinct *GATA1* mutations (Refs 30, 41, 61). Indeed, it has been suggested that aneuploidy can promote genomic instability, which has been proven in yeast (Refs 62, 63). These observations suggest an increased mutation rate of haematopoietic cells of DS fetuses, which could contribute to their higher risk of leukaemia. We have recently tested this hypothesis by whole genome sequencing (WGS) of clonally expanded single haematopoietic stem and progenitor cells (HSPCs) of non-DS and DS human fetuses (Ref. 20). We have shown that HSPCs of DS fetuses have 34 extra somatic mutations compared with HSPCs of non-DS fetuses (Ref. 20). This might not seem a lot, however, non-DS fetal HSPCs have already shown a somatic mutation rate of 100 base substitutions per year, which is 5.8 times higher compared with the mutation rate of adult HSPCs (Ref. 20). This relatively high prenatal mutation rate may contribute to the increased leukaemia risk in children compared with young adults by increasing the chance of acquiring an oncogenic mutation (Ref. 64). The even higher mutation load in HSPCs of DS fetuses could further increase this chance.

### Underlying causes of the increased mutation load in DS fetal cells

The driving mechanisms behind the increased somatic mutation load observed in fetal DS stem cells are still unknown (Ref. 20). Increased reactive oxygen species (ROS) production, caused by oxidative stress, has been found in almost all cancer types and is thought to be an important driving factor for tumour development (Refs 65, 66). Deficiencies in mitochondrial functioning can result in increased ROS production (Ref. 67). Several cell types of DS individuals show mitochondrial dysfunction and increased levels of ROS (Refs 68–73). Moreover, several genes involved in mitochondrial function are located on chromosome 21 and overexpressed in DS, which further supports deregulation of mitochondrial function (Refs 74–76). However, knowledge on an association between increased ROS levels and DS-leukaemogenesis is lacking.

**Fig. 1.**
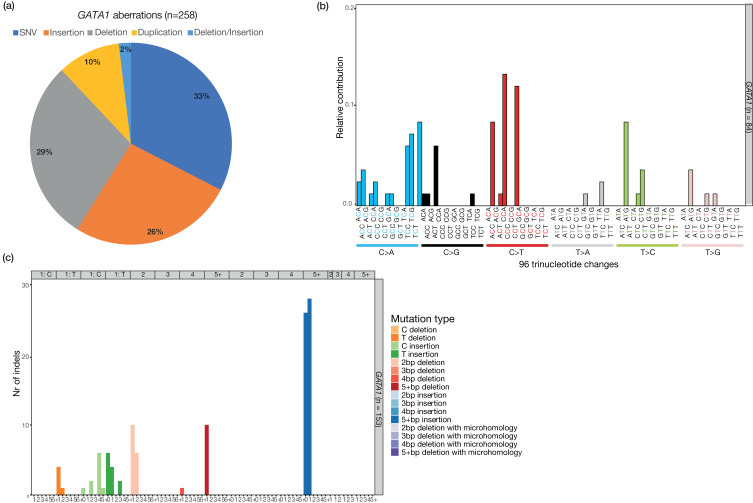
*GATA1* aberrations in transient abnormal myelopoiesis (TAM). Analysis of all *GATA1* mutations identified in TAM, which are annotated in the Catalogue of Somatic Mutations in Cancer (COSMIC) Cancer Gene Census database and *GATA1* mutations from the recently published study of Labuhn *et al*. (Refs 30, 31, 41, 61, 78, 138–149). (a) Pie chart showing the different types of *GATA1* mutations observed in TAM (pre)leukaemic blasts. (b) 96-trinucleotide spectrum of all single nucleotide variants (SNVs) observed in *GATA1*. (c) The spectrum of insertions and deletions (indels) within *GATA1.*

Oxidative stress can damage DNA, which can result in mutations if left unrepaired (Ref. 77). ROS are mainly mutagenic by inducing 8-oxoguanine lesions, which increase mispairing with adenine instead of cytosine (Ref. 77). G:C > T:A mutations were identified as the predominant base substitution type causing mutations in *GATA1* (Fig. 1b) (Ref. 78). These predominant G:C > T:A mutations could indicate that ROS production induces *GATA1* mutations in DS. Mutational processes, such as oxidative stress, are known to generate a characteristic pattern of mutations, which is called a mutational signature (Ref. 79). These signatures are classified in the Catalogue of Somatic Mutations in Cancer (COSMIC) database (Ref. 80). COSMIC signature 18, which has previously been associated with oxidative stress mutagenesis (Ref. 81), was identified in a subset of HSPCs isolated from DS fetuses (Ref. 20). However, this signature did not contribute to the somatic mutations observed in the (pre)leukaemic blasts of TAM patients (Ref. 20) or to the single base substitutions causing GATA1s in TAM (Fig. 1b). Instead, insertions, deletions and duplications are more often the cause of GATA1s in TAM (Fig. 1a). The indel profile of the identified *GATA1* mutations in TAM (pre)leukaemic blasts shows that 5 + base pair insertions are the most frequently observed type of indels causing GATA1s (Fig. 1c). The *GATA1* indel profile does not show similarity to a known small indel COSMIC signature (Ref. 80). Therefore, the mutational process causing this specific indel profile is not yet known and needs further investigation (Fig. 1c). Altogether, there is evidence of mitochondrial dysfunction and increased levels of ROS in DS. However, DNA damage caused by ROS is not necessarily needed to induce the somatic mutations that are necessary for the development of TAM (Ref. 20).

Besides mitochondrial dysfunction, deficiencies in DNA damage repair may also explain the increased somatic mutation load observed in DS fetal cells (Refs 20, 82). Multiple studies have shown a defect in DNA repair in DS cells (Refs 78, 82). Also, in the fetal liver, lower base excision repair activity was detected in DS-tissues compared with non-DS tissues (Ref. 78). These findings support the hypothesis that, already in the prenatal setting, ineffective DNA damage repair in DS could, together with mitochondrial dysfunction, lead to increased mutagenesis and potentially to phenotypic diversity.

### Epigenetic and transcriptional alterations in DS haematopoiesis

Besides genetic aberrations, epigenetic changes can contribute to the inheritable phenotypic diversity within cell populations by changing transcription levels. Many studies have shown genome-wide epigenetic alterations in cells of DS fetuses prior to leukaemia development, not solely restricted to chromosome 21 (Refs 29, 83–85). Transcriptome analysis of fetal fibroblasts isolated from monozygotic twins, discordant for T21, showed that differential expression is organised in chromosomal domains that vary in size and contain up to 507 genes (Ref. 29). These well-defined domains that consist of neighbouring genes sharing differential expression profiles are called gene expression dysregulation domains (GEDDs) (Ref. 29). Syntenic blocks along the mouse chromosomes showed that these GEDDs were conserved in induced pluripotent stem cells (IPSCs) derived from fetal fibroblasts and fibroblasts from the Ts65Dn DS mouse model in which DS is modelled through a partial trisomy comprised of a distal portion of mouse chromosome 16 and a centromeric portion of mouse chromosome 17 (Ref. 29). This finding indicates a consistent influence of T21 on the transcriptome (Ref. 29). The identified GEDD pattern in mice and human IPSCs is shown to be associated with chromatin modifications in T21 cells, since the altered H3K4me3 profile observed in these cells followed the GEDD pattern (Ref. 29). H3K4me3 marks are positively correlated with these gene expression levels, suggesting that the transcriptomic alterations in DS are a result of chromatin modifications (Ref. 29). Several genes encoding chromatin modifiers, such as *HMGN1*, *DYRK1A*, *BRWD1* and *RUNX1*, are located on chromosome 21 and may explain the altered H3K4me3 profile (Ref. 29). In line with this, B-cells from *HMGN1* overexpressing mice show a global increase in hyperacetylation of histone H3K27 (Ref. 86). This hyperacetylation also causes transcriptional changes, which recapitulates the transcriptional changes observed in pro-B-cells of the Ts1Rhr DS mice model in which DS is modelled by triplication of 31 genes on a mouse chromosome orthologous to the DSCR (Ref. 86). Overexpression of several genes that encode chromatin modifiers, located on chromosome 21, may be responsible for global transcriptional changes observed in DS cells.

Characterisation of DNA methylation profiles of DS fetuses exhibited inconsistent results, however, these all demonstrated an aberrant methylation pattern in DS newborns (Refs 83–85, 87–89). Further research is warranted to clarify the role of these changes in the development of leukaemia in DS.

These findings indicate that besides genetic aberrations, epigenetic alterations can be found in DS cells, which contribute to the increased inheritable phenotypic diversity. However, additional oncogenic mutations and/or epigenetic alterations are needed to drive leukaemia in DS. The chance to acquire an oncogenic mutation causing leukaemia in DS may be increased by the enlarged phenotypic diversity that is already present early during development.

### Epigenetic and transcriptional differences in DS-associated leukaemias

RNA expression profiles of TAM and ML-DS have shown to be similar to each other, and distinct from non-DS AMKL (Refs 30, 85, 90). However, RNA expression levels of recurrently mutated genes in ML-DS, such as *RAD21* and *EZH2*, appeared to be similar in ML-DS and non-DS AMKL patients (Ref. 30). Yet, there are some exceptions. Genome-wide DNA methylation analysis demonstrated that mononuclear cells of both TAM and ML-DS are epigenetically deregulated (Ref. 85). Malinge *et al*. showed that mutations in *GATA1* in TAM led to focal DNA hypermethylation of specific target genes, whereas the DSCR remained hypomethylated (Ref. 85). The hypermethylated target genes are related to haematological development and regulation of key cellular processes, such as proliferation, growth, cell cycle regulation and cell death (Ref. 85). In addition, ML-DS and TAM have a similar DNA methylation pattern (Ref. 85). The strong similarities in gene expression and DNA methylation patterns between TAM and ML-DS suggest that the transient nature of TAM and the later development of ML-DS are unlikely to be driven by changes in epigenetic factors. However, mononuclear cells from ML-DS patients were hypomethylated compared with mononuclear cells from non-DS AMKL patients, indicating that ML-DS is epigenetically different from non-DS AMKL (Ref. 85).

RNA expression profiles of DS-ALL cases are very heterogeneous. Some DS-ALL cases cluster together, whereas other cases are more similar to non-DS ALL subtypes, such as the *BCR-ABL1* translocated type (Refs 51, 91). In line with this, there are no genes on chromosome 21 that show a significantly higher expression in DS-ALL when compared with non-DS ALL (Ref. 91). Cases with increased *CRLF2* expression are found to have a Philadelphia-like expression signature (Ref. 91). Nevertheless, the fusion created by the Philadelphia chromosome, *BCR-ABL1*, is uncommon in DS-ALL (Refs 91, 92). The heterogeneity of DS-ALL patients is further supported by the DNA methylation profile, which shows clustering per subtype as opposed to DS-ALL clustering together (Ref. 91). However, in most subtypes, the *RUNX1* promoter is hypermethylated in DS-ALL compared with non-DS ALL (with exception of *ETV6-RUNX1* non-DS ALL) (Ref. 91). Also, in DS individuals without leukaemia, this promoter is, already congenitally, found to be hypermethylated (Ref. 91). The fact that *RUNX1* is essential for B-cell differentiation suggests that *RUNX1* hypermethylation is a predisposing factor for BCP-ALL in DS individuals (Ref. 91). Strikingly, another study demonstrated that DS-ALL and non-DS ALL are distinguishable by the overexpression of genes, marked with H3K27me3 (Ref. 93). This study showed that H3K27me3 marks are globally suppressed in B-cells of the TsRhr1 DS mice model, which is possibly explained by overexpression of *HMGN1* (Ref. 93). This suppression, in combination with H3K4me3, leads to overexpression of genes that contribute to B-cell proliferation in Tshr1, pointing out the importance of epigenetic changes in DS-ALL (Ref. 93).

An extra chromosome 21 in DS individuals serves as the basis of a broad variety of genetic and epigenetic changes. As mentioned before, we have previously demonstrated that fetal HSPCs of DS fetuses have an increased somatic mutation load compared with HSPCs of non-DS fetuses (Ref. 20). Using WGS data of fetal HSPCs, we have constructed a model to determine the mutation rate during fetal haematopoeisis (Ref. 20). For example, HSPCs of a 14 WG (weeks of gestation) DS fetus have 58 somatic mutations (95% confidence interval 40.16–75.39). When we use our model to extrapolate the mutation load for a non-DS fetus of the same gestational age, these HSPCs would have 24 (95% confidence interval 11.88–34.8) somatic mutations. This suggests that HSPCs of a 14 WG DS fetus have a 2.4-fold increase in somatic mutations (Ref. 20). This increased mutation load combined with the 3.5 times higher number of HSPCs (Ref. 94) indicates that DS fetal HSPCs have a higher risk to acquire an oncogenic mutation during fetal haematopoiesis compared with non-DS fetal HSPCs. However, the specific time-based higher incidence of AML and BCP-ALL in these children, with a lower risk of solid tumours (Ref. 10), suggests that other factors may play a role. Additional epigenetic aberrations on top of the increased mutation load further increase the phenotypic diversity, but still do not completely explain the increased incidence of leukaemia in DS children. In conclusion, these findings support our hypothesis that the increased risk of leukaemia in DS children is not solely explained by phenotypic diversity.

## Selection dynamics

According to the somatic evolutionary model, oncogenic clones can be positively selected depending on the tissue context (Ref. 95). Alterations in the haematopoietic microenvironment, such as changes in the cellular composition of the niche as well as the immune system, which are observed in DS, are likely to affect selection dynamics contributing to the progression of leukaemogenesis.

### The fetal liver niche in DS

During fetal development, the liver is the dominant site of haematopoiesis, which transfers to the bone marrow after birth (Ref. 96). As discussed before, the first oncogenic mutations in DS are thought to be acquired during fetal development (Ref. 2). These observations suggest that the fetal liver microenvironment, according to the somatic evolutionary model, may have a crucial role in the selection and growth of (pre)leukaemic clones that arise during fetal development in DS. Indeed, it has been shown that megakaryocyte progenitors isolated from the yolk sac or fetal liver of GATA1s mice show a hyperproliferative phenotype (Ref. 40). Notably, this effect was transient, indicating that GATA1s has a developmental stage-dependent effect (Ref. 40). Little is known about which cells within the human fetal liver support the expansion and differentiation of HSPCs during fetal haematopoiesis. Mice studies have shown that fetal liver stromal cells and Nestin + NG2 + pericytes support the proliferation of HSPCs (Refs 97–99). Moreover, fetal liver stromal cells support the expansion of megakaryocyte committed progenitors (Ref. 100).

Both supportive cell types express insulin growth factor-2 (*IGF2*), which is known to promote the expansion of HSPCs during fetal haematopoiesis (Refs 97–99). More importantly, the proliferation of mice fetal liver megakaryocyte progenitors is reliant on IGF signalling, whereas proliferation of adult megakaryocyte progenitors isolated from bone marrow is not (Ref. 35). Several studies have demonstrated that IGF signalling is increased in DS (Refs 35, 101). Recently, it has been shown that increased IGF signalling is associated with the overproduction of CD43+ haematopoietic progenitors derived from T21 IPSCs (Ref. 101). CD43 is the earliest marker of full haematopoietic commitment after endothelial to haematopoietic transition, which suggests that T21 promotes this transition (Ref. 101). Furthermore, T21 CD43+ haematopoietic progenitors are more sensitive to inhibition of IGF signalling compared with CD43+ haematopoietic progenitors in which the extra chromosome 21 is silenced by site-directed insertion of the X-inactive inactive specific transcript (*XIST*) gene in one copy of chromosome 21 (Ref. 101). Altogether, these findings suggest that T21 itself increases IGF signalling (Ref. 101).

IGF signalling seems to have a preserved role in the proliferation of TAM and ML-DS (pre)leukaemic blasts (Ref. 35). Klusmann *et al*. (Ref. 35) showed that the IGF receptor 1 (*IGFR1*) is expressed higher in primary ML-DS leukaemic blasts and ML-DS cell lines, compared with non-DS AMKL leukaemic blasts and cell lines. The same study demonstrated that inhibition of IGF signalling results in impaired proliferation of TAM and ML-DS (pre)leukaemic blasts (Ref. 35). Moreover, overexpression of *IGF2* in GATA1s liver mice megakaryocyte progenitors resulted in increased proliferation, whereas a negligible effect was observed in *GATA1* wild-type liver megakaryocyte progenitors and GATA1s bone marrow megakaryocyte progenitors (Ref. 35). These findings suggest that there might be an interaction between IGF signalling and *GATA1* in the fetal liver. It has been shown that the IGF signalling pathway activates *E2F* target genes through activation of mTOR signalling and upregulation of *MYC* (Ref. 35). *E2F* target genes play a role in the regulation of cell proliferation (Ref. 102). Normally, GATA1 represses the expression of *E2F* target genes by direct interaction with E2F transcription factors and via repression of MYC, this will result in a decrease in cell proliferation (Ref. 35). However, GATA1s is not able to interact with E2F transcription factors and thereby cannot regulate the expression of *E2F* target genes, which results in overactive IGF signalling and hyperproliferation (Refs 35, 36). Together, these findings support that increased IGF signalling in DS has a role in the selection and proliferation of (pre)leukaemic TAM clones within the fetal liver. The role of IGF signalling in the development of DS-ALL has, to our knowledge, not been studied yet. Intriguingly, in non-DS paediatric BCP-ALL, it has been demonstrated that IGF signalling promotes the expansion of leukaemic blasts and therefore IGF signalling might also have a role in the development of DS-ALL (Refs 103, 104). Another study found granulocyte-macrophage colony-stimulating factor (GM-CSF), secreted by fetal liver stromal cells, as the main growth factor supporting TAM (pre)leukaemic blasts (Ref. 105). Here, IGF signalling was not identified to have a supportive role in the growth of TAM (pre)leukaemic blasts (Ref. 105).

Altogether, these studies indicate that the fetal liver niche cells and IGF signalling pathway in DS contribute to the proliferation and selection of pre(leukaemic) blasts. This supports the hypothesis that alterations in the fetal liver microenvironment in DS can change selection dynamics in such a way that *GATA1*-mutated clones have a growth advantage, whereas the bone marrow does not provide this advantage. This model may explain why TAM spontaneously disappears when haematopoiesis migrates to the bone marrow after birth (Refs 13, 14). In turn, the additional oncogenic mutations observed in ML-DS leukaemic blasts may subsequently increase the phenotypic diversity among cells (Refs 30, 41). This increased phenotypic diversity will then result in the selection of the leukaemic clones and progression to ML-DS.

### Chronic inflammation and interferon (IFN) signalling in DS

According to the immune surveillance theory, the immune system acts as a barrier to prevent cancer development (Refs 106, 107). However, sequencing studies have demonstrated a neutral drift for most missense and nonsense mutations in normal and cancer tissues, suggesting that selection or removal of these mutations is a stochastic process (Ref. 108). Of note, not all missense mutations result in a neoantigen. This finding suggests that immune cells may not have a crucial role in the first steps of cancer initiation (Ref. 108). Several studies have demonstrated that inflammation changes the selection pressure on oncogenic clones (Refs 109–111). Not surprisingly, chronic inflammation is a hallmark of cancer (Ref. 112). In addition, chronic inflammation can result in genomic instability (Ref. 113), which increases the chance to acquire oncogenic driver mutations.

Abnormalities in immune function are a common characteristic of DS (Ref. 114). DS individuals have an increased incidence of autoimmune diseases and infections (Ref. 114). This observation suggests that tissues of DS individuals show signs of chronic inflammation, which may contribute to the increased leukaemia risk. Indeed, inflammatory factors related to chronic inflammation are upregulated in blood cells and plasma of children and adults with DS (Refs 115–118). NK-cells and T-cells are hyperactivated, whereas cells of the myeloid compartment show signs of inflammation and increased cytotoxic potential (Refs 117, 119). Moreover, several studies have indicated that the blood cell composition is perturbed in DS, which further supports the deregulation of the immune system (Refs 94, 120, 121). The number of HSCs and megakaryocyte and erythrocyte progenitors are increased in the DS fetal liver, whereas the number of granulocyte–macrophage progenitors, B-cells, pre–pro-B cells and pro-B cell progenitors are decreased (Refs 94, 120, 121). The observed depletion of B-cells, pre–pro-B cells and pro-B cells suggests a maturation defect of B-cells during DS fetal haematopoiesis. Studies in adults have shown that this depletion was preserved over time (Refs 117, 122).

Altogether, these findings suggest that the haematopoietic system of both children and adults with DS is altered and shows signs of chronic inflammation. However, if an altered immune system and chronic inflammation contribute to the increased incidence of leukaemia in children with DS, it remains unclear why this risk decreases tremendously during adolescence, since alterations of the immune system are preserved during adulthood. Moreover, it remains an open question why this increased risk is limited to the haematopoietic system because chronic inflammation is associated with increased cancer risk in general and DS individuals do not experience an increased risk of other malignancies besides paediatric leukaemia (Refs 10, 112).

Over the last years, it has become evident that hyperactive immune signalling can induce changes in the haematopoietic system, such as the increased proliferation of HSPCs and a biased differentiation towards the myeloid lineage (Ref. 123). Overactive IFN signalling is associated with increased proliferation of fetal and adult HSCs and myeloid skewing in adults (Refs 124–128). Four of the six IFN receptors are located on chromosome 21 and proteomics and RNA sequencing approaches have shown consistent activation of IFN signalling in immune cells of adults with DS (Refs 115, 117, 118). These findings suggest that overactive IFN signalling may contribute to the observed changes in DS haematopoiesis. On the contrary, it has been shown that IFN signalling has an antiproliferative effect on megakaryocyte progenitors, which are enriched in DS compared with karyotypically normal newborns (Refs 129, 130). IFN signalling is increased in adult bone marrow megakaryocyte progenitors isolated from wild-type mice compared with megakaryocyte progenitors isolated from the fetal liver (Ref. 129). Indeed, both bone marrow GATA1s and GATA1 wild-type megakaryocyte progenitors, isolated from *IFNAR1*−/− mice, show a hyperproliferative phenotype (Ref. 129). However, hyperproliferation was more pronounced in GATA1s *IFNAR1*−/− mice (Ref. 129). In line with this, IFN-alpha stimulation resulted in a more pronounced decrease in proliferation of GATA1s megakaryocyte progenitors as compared with GATA1 wild-type megakaryocyte progenitors (Ref. 129). Taken together, these findings imply that there is an interplay between GATA1s and IFN signalling, which may contribute to the proliferation and selection of (pre)leukaemic blasts in the fetal liver and the spontaneous remission of TAM (Ref. 129). Indeed, (pre)leukaemic blasts from TAM patients show a higher expression of IFN signalling genes, which is, considering the anti-proliferative effect, consistent with the spontaneous remission of TAM within three months after birth (Ref. 129). Nonetheless, leukaemic blasts from ML-DS patients also highly express IFN signalling genes, indicating that additional acquired oncogenic mutations in ML-DS are needed for leukaemic progression (Ref. 129).

## Discussion

**Fig. 2.**
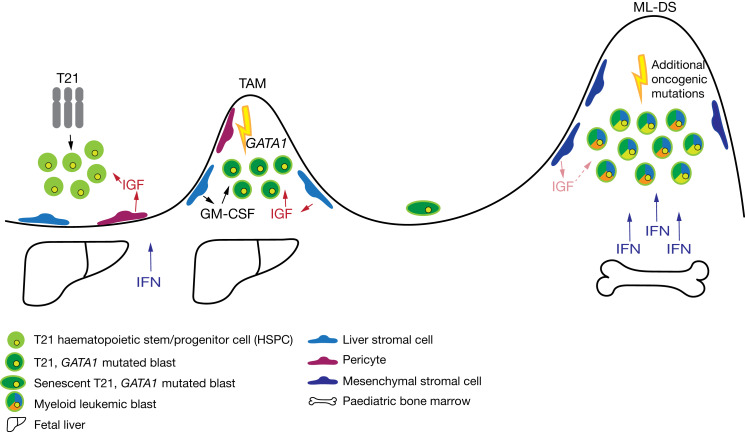
Development of transient abnormal myelopoiesis (TAM) and myeloid leukaemia of Down syndrome (ML-DS). Model for the development of TAM and ML-DS in Down syndrome individuals. Schematic representation, indicating the initiation of TAM before birth when trisomy 21 (T21) fetal haematopoietic stem and progenitor cells (HSPCs) acquire a *GATA1* mutation. The proliferation of (pre)leukaemic TAM blasts in the fetal liver is supported by secretion of granulocyte-macrophage colony-stimulating factor (GM-CSF) in combination with increased insulin growth factor-2 (IGF) signalling and lower levels of IFN signalling. TAM resolves when haematopoiesis migrates to the bone marrow after birth and interferon (IFN) signalling increases. Additional oncogenic mutations in an existing *GATA1* mutated clone provide these cells with an additional growth advantage and are required for progression towards ML-DS.

**Fig. 3.**
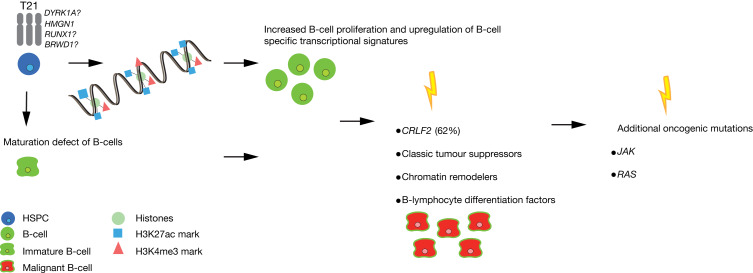
Development of DS acute lymphoblastic leukemia (DS-ALL). Model for the development of acute lymphoblastic leukemia (ALL) in Down syndrome individuals. Trisomy 21 (T21) haematopoietic stem and progenitor cells (HSPCs) show a B-cell maturation defect. An extra copy of genes encoding chromatin modifiers on chromosome 21 causes a decrease in H3k27me3 marks and an increase in H3K27ac and H3K4me3 marks on genes involved in B-cell proliferation and B-ALL development, which will result in overexpression of these genes. The acquisition of cancer driver mutations and additional oncogenic mutations is sufficient for the development of DS-ALL.

Children with DS show an increased incidence of AMKL and BCP-ALL during the first years of life (Ref. 9). However, the reason for this remains unknown. It is well accepted that the presence of a constitutive trisomy of chromosome 21 is sufficient to perturb (fetal) haematopoiesis (Refs 6, 94, 101, 120, 121). Interestingly, complete or partial gains of chromosome 21 are frequently seen in non-DS paediatric B-ALL cases and non-DS AMKL, but are rarely observed in adult leukaemias (Refs 27, 57, 131). Nonetheless, besides T21, additional oncogenic mutations in somatic T21 and DS-associated leukaemias are needed to drive leukaemogenesis (Figs. 2 and 3). These observations suggest that trisomy of chromosome 21 may prime the haematopoietic system for cancer and that DS-associated leukaemia could be used to study paediatric leukaemia in general (Figs. 2 and 3).

In adult cancers, the acquisition of oncogenic mutations is thought to be rate-limiting (Ref. 132). This may explain why aging is the biggest risk factor for developing cancer, since somatic mutations gradually increase throughout life (Refs 20, 22, 25, 132). However, the incidence of leukaemia in young children compared with (young) adults is higher even though their cells harbour less somatic mutations (Ref. 133). Remarkably, DS children show an even higher incidence (Refs 3, 7, 8). Together, this suggests that paediatric leukaemia, in general, has a different aetiology compared with adult leukaemia.

It has been shown that genetic drivers of childhood leukaemia are acquired prenatally (Ref. 64). The increased somatic mutation rate of fetal HSPCs and the even higher mutation load of DS fetal HSPCs compared with the rate of adult HSPCs may partially explain the higher incidence of leukaemia in (DS) children (Refs 20, 22). This increased mutation rate promotes the phenotypic diversity of fetal HSPCs and the chance to acquire an oncogenic mutation (Refs 20, 22). Of note, the postnatal somatic mutation rate of DS-HSPCs has not been characterised yet, whereas additional mutations required for ML-DS and possibly also for DS-ALL are acquired after birth. Since the risk of leukaemia in DS decreases tremendously during life (Ref. 8), we suggest that other factors, such as selection, play an additional role in the development of paediatric leukaemia and DS-associated leukaemogenesis.

Several findings suggest that, besides the observed increased phenotypic diversity, changes in selection dynamics in the fetal liver and bone marrow may have a role in leukaemic development in non-DS and DS children. First of all, the (pre)leukaemic TAM blasts spontaneously disappear when haematopoietic cells migrate to the bone marrow, suggesting that the selection dynamics in the bone marrow are different from the fetal liver (Refs 13, 14). Second, the mutational landscape of childhood leukaemia, in general, is different from adult leukaemia (Refs 131, 134). Also, cancer driver mutations found in DS-associated leukaemia are less frequently found in non-DS-associated leukaemia (Refs 16, 17, 30, 49, 53). These observations imply that different selection forces act on oncogenic clones (Refs 131, 134). *GATA1* mutations are only observed in non-DS AMKL patients with a somatic T21, suggesting that T21 is needed to select these (pre)leukaemic clones (Refs 49, 135, 136). Remarkably, germline *GATA1* mutations without a T21 background are associated with many rare red cell disorders (Ref. 137), which points towards a specific aetiology of DS and T21-associated leukaemogenesis. This distinct aetiology and selection raise the question if a model based on DS leukaemogenesis would be applicable to study paediatric leukaemia in general.

In conclusion, we suggest, in line with a somatic evolutionary point of view, that leukaemogenesis in both non-DS and DS-associated leukaemia is driven by phenotypic diversity and changes in selection dynamics. However, both phenotypic diversity and selection dynamics are likely different in non-T21 associated leukaemia and DS-associated leukaemia. These differences suggest that non-T21 associated leukaemia has a different aetiology. Here, we propose a model in which leukaemic development in DS is driven by an increase in inheritable phenotypic diversity of HSPCs, caused by epigenetic changes and an increased somatic mutation rate during fetal development, which increases the chance to acquire an oncogenic hit. In turn, leukaemogenesis is further promoted by the selection of (pre)leukaemic clones in the fetal liver and paediatric bone marrow niche. This model would mean that treatment of DS-leukaemogenesis should possibly not only focus on genetic and epigenetic changes, but also on the microenvironment that supports the selection of leukaemic clones. This offers opportunities for future therapy development.
